# E3 Ubiquitin Ligase APC/C^Cdh1^ Regulation of Phenylalanine Hydroxylase Stability and Function

**DOI:** 10.3390/ijms21239076

**Published:** 2020-11-28

**Authors:** Apoorvi Tyagi, Neha Sarodaya, Kamini Kaushal, Arun Pandian Chandrasekaran, Ainsley Mike Antao, Bharathi Suresh, Byung Ho Rhie, Kye Seong Kim, Suresh Ramakrishna

**Affiliations:** 1 Department of Biomedical Science, Graduate School of Biomedical Science and Engineering, Hanyang University, Seoul 04763, Korea; apoorvityagi09@gmail.com (A.T.); neyha19@gmail.com (N.S.); kaminikaushal10@gmail.com (K.K.); aruask.iq@gmail.com (A.P.C.); ainsleyantao@gmail.com (A.M.A.); (bharathi.suri@gmail.com (B.S.); bhrhie0712@hanmail.net (B.H.R.); 2 College of Medicine, Hanyang University, Seoul 04763, Korea

**Keywords:** enzyme assay, hyperphenylalaninemia, liver cancer, neurological damage, tetrahydrobiopterin, ubiquitin-proteasome system

## Abstract

Phenylketonuria (PKU) is an autosomal recessive metabolic disorder caused by the dysfunction of the enzyme phenylalanine hydroxylase (PAH). Alterations in the level of PAH leads to the toxic accumulation of phenylalanine in the blood and brain. Protein degradation mediated by ubiquitination is a principal cellular process for maintaining protein homeostasis. Therefore, it is important to identify the E3 ligases responsible for PAH turnover and proteostasis. Here, we report that anaphase-promoting complex/cyclosome-Cdh1 (APC/C)^Cdh1^ is an E3 ubiquitin ligase complex that interacts and promotes the polyubiquitination of PAH through the 26S proteasomal pathway. Cdh1 destabilizes and declines the half-life of PAH. In contrast, the CRISPR/Cas9-mediated knockout of *Cdh1* stabilizes PAH expression and enhances phenylalanine metabolism. Additionally, our current study demonstrates the clinical relevance of PAH and Cdh1 correlation in hepatocellular carcinoma (HCC). Overall, we show that PAH is a prognostic marker for HCC and Cdh1 could be a potential therapeutic target to regulate PAH-mediated physiological and metabolic disorders.

## 1. Introduction

Phenylalanine hydroxylase (PAH) is a highly regulated liver enzyme that plays a key role in phenylalanine catabolism. Mammalian PAH is tetrameric, and its 52 kDa subunits are composed of n-terminal regulatory domain for allosteric activation by Phenylalanine (Phe), a central catalytic domain, and c-terminal helix responsible for tetramer formation [1,2] PAH catalyzes the hydroxylation of l-phenylalanine (l-Phe) into l-tyrosine (l-Tyr) using tetrahydrobiopterin (BH4) and molecular oxygen [3,4], which is the initial step in governing the synthesis of catecholamines, thyroxin, and melatonin [5]. Under a non-pathological state, PAH maintains physiological levels of l-Phe (<120 µM) by the degradation of excessive intracellular l-Phe [6]. Mutations or deficiency in the PAH gene leads to hyper-accumulation of l-Phe, causing phenylketonuria (PKU) in different ethnic populations and the depletion of precursors involved in neurotransmitter biosynthesis in the central nervous system that overall results in loss of cognitive ability and neurological disorders [7]. Toxic accumulation of phenylalanine in the blood and brain is one of the clinical manifestations in patients with PKU or hyperphenylalaninemia [8]. Over 1000 mutations have been identified in the *PAH* gene, and according to the Locus Knowledgebase (PAHvdb, www.pahdb.mcgill.ca), about 60% of these mutations are missense mutations that change the conformation of the enzyme, thus altering its ability to bind to the l-Phe substrate and BH4 cofactor [3,4]. Understanding the regulatory properties of PAH is, therefore, essential to determine the pathophysiological mechanisms in PKU and delineate novel therapeutic strategies. Moreover, the expression profiles of most of the liver enriched genes in hepatocellular carcinoma (HCC) showed downregulation behavior in tumor tissues than that of non-tumor tissues. Interestingly, high expression of these liver enriched genes showed prolonged overall survival suggesting favorable prognostic effects in HCC [9]. In addition to the covalent and non-covalent regulation of kinetic properties, the amount of available enzyme in cells also plays a vital role [10].

Post-translational modifications such as ubiquitination, deubiquitination, and sumoylation could be a critical factor in the regulation of PAH turnover and its corresponding enzymatic activity in cells. Protein degradation mediated by ubiquitination is one of the most important cellular processes for maintaining protein levels, particularly for enzymes that regulate several cellular functions. Protein ubiquitination is an important pathway where the sequential activation of ubiquitin by ubiquitin-activating enzyme (E1), ubiquitin conjugases (E2), and ubiquitin ligases (E3) transfer ubiquitin molecules to target substrates for mono- or poly-ubiquitination [11,12]. Thus, finding a specific E3 ligase for PAH might help us to understand the molecular mechanism of its dynamics at the physiological level.

In this study, we demonstrate that PAH undergoes anaphase-promoting complex/cyclosome (APC/C) dependent proteolysis via Cdh1. The ubiquitin ligase APC/C plays essential roles in and outside the eukaryotic cell cycle and is the most complex molecular machine that catalyzes ubiquitination reactions [13]. The enzymatic activity of the APC/C complex depends on its association with a coactivator, Cdh1 that binds directly to the APC/C core complex and activates E3 ubiquitin ligase activity, thereby contributing to its substrate recognition and specificity. The present study aims to investigate the role of E3 ligase APC/C^Cdh1^ on PAH ubiquitination and enzymatic activity. Additionally, we investigate whether APC/C^Cdh1^-mediates the turnover of PAH and its association with the prognosis of patients with HCC.

## 2. Results

### 2.1. PAH Is Degraded by the 26S Proteasomal Pathway

**Figure 1:**
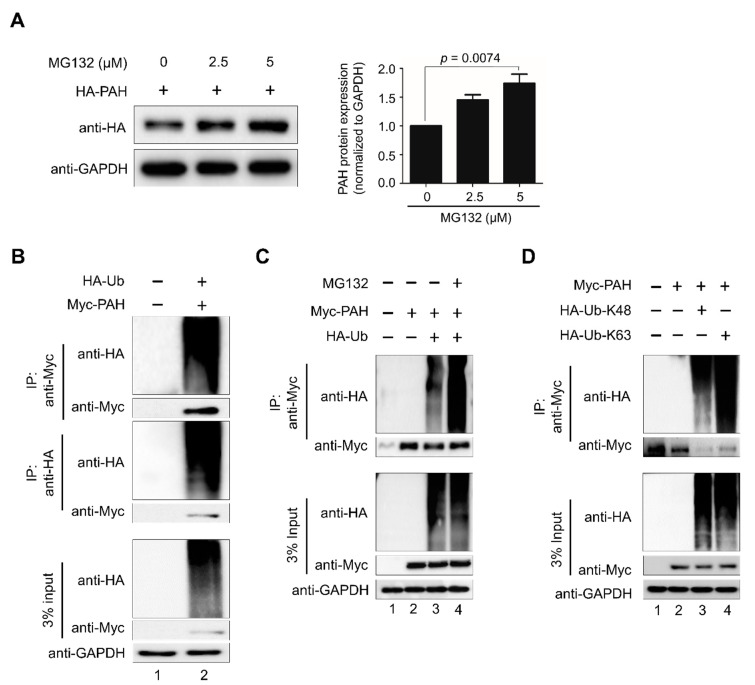
PAH is degraded by the 26S proteasomal pathway. (**A**) HEK293 cells were transfected transiently with HA-PAH and treated with increasing concentrations of MG132 and analyzed by Western blotting with the indicated antibodies. Band intensity was estimated using ImageJ software, normalized with GAPDH, and represented graphically. The data shown here are the mean ± SD of three independent experiments. One-way ANOVA followed by Tukey’s post hoc test was used, and *p*-values are indicated. (**B**) HEK293 cells were transfected with Myc-PAH along with HA-ubiquitin and immunoprecipitated with Myc or HA antibodies and immunoblotted with indicated antibodies. (**C**) Effect of MG132 on the ubiquitination and proteasomal degradation of Myc-PAH was determined. HEK293 cells were transfected with Myc-PAH along with HA-ubiquitin and immunoprecipitated with Myc antibody and immunoblotted with indicated antibodies. (**D**) K48- and K63-linked polyubiquitination of PAH. HEK293 cells were transfected with Myc-PAH along with HA-Ub-K48 and HA-Ub-K63 and immunoprecipitated with Myc antibody and immunoblotted with indicated antibodies.

Understanding the importance of PAH protein turnover in maintaining a stable pool of l-Phe and l-Tyr at physiological condition, we investigated whether PAH undergoes degradation via the 26S proteasomal pathway. To this end, HA-PAH was transfected ectopically in HEK293 cells and treated with increasing concentrations of proteasomal inhibitor MG132. The effect of MG132 on the proteasomal degradation of PAH following incubation with different concentrations of inhibitor was examined after 48 h. Our results reveal that MG132 blocks 26S proteasome activity, and thus PAH protein get accumulated in the cell in a dose-dependent manner at all levels tested (Figure 1A). Next, to investigate the ubiquitination of PAH, we co-transfected Myc-tagged-PAH and HA-tagged-ubiquitin in HEK293 cells. Immunoprecipitation analysis with the antibodies indicated were then performed to further validate the in vivo ubiquitination of PAH. The ubiquitin smear was observed only in the transfected PAH samples along with the ubiquitin construct, which indicated that PAH protein undergoes polyubiquitination and degradation (Figure 1B, lane 2). Treatment with proteasome inhibitor MG132 on PAH ubiquitination resulted in increased accumulation of ubiquitinated PAH protein in HEK293 cells (Figure 1C, lane 4) when compared with MG132 untreated samples (Figure 1C, lane 3). Thus, our results demonstrate that PAH undergoes degradation via the 26S proteasomal pathway.

We next investigated whether PAH undergoes K48- or K63-linked polyubiquitination using mutant ubiquitin constructs in which all the lysine residues were replaced by arginine residues, except at the Lys-48 or Lys-63 position. Thus, we co-transfected HA-ubiquitin-K48 or HA-ubiquitin-K63 and Myc-PAH in HEK293 cells and performed immunoprecipitation with anti-Myc antibody, followed by Western blot with anti-HA and anti-Myc antibodies (Figure 1D). Interestingly, our data showed a characteristic high molecular weight ubiquitination smear on PAH with both K48- and K63-linked mutant ubiquitin, indicating that PAH adopts both K48- and K63-linked topology for the proteasome linked degradation of PAH (Figure 1D, lanes 3 and 4).

### 2.2. APC/C^Cdh1^ Is the E3 Ligase Responsible for PAH Protein Degradation

**Figure 2:**
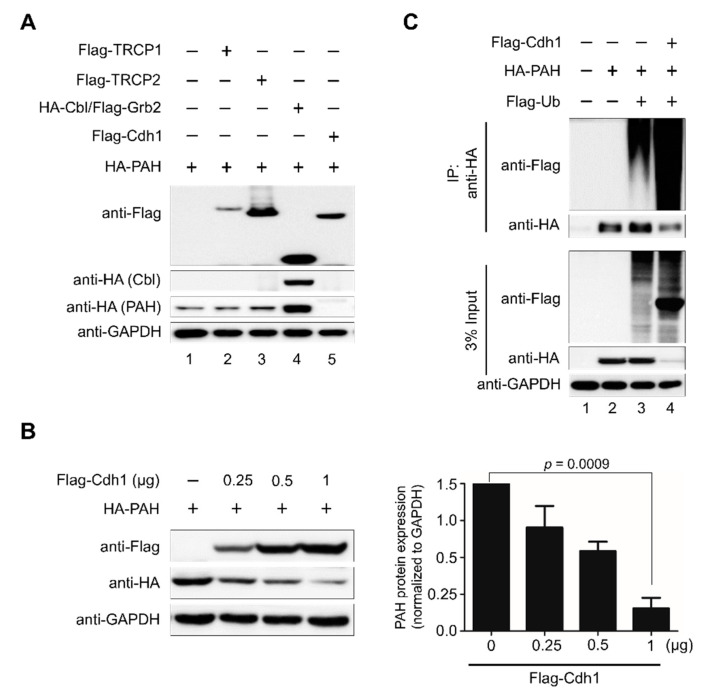
APC/C^Cdh1^ is the E3 ligase responsible for PAH protein degradation. (**A**) HEK293 cells were transfected with different E3 ligases along with ectopically expressing HA-PAH. Downregulation of PAH was analyzed by Western blotting. GAPDH was kept as a loading control. (**B**) HEK293 cells were transfected with a constant amount of HA-PAH (1 µg) along with increasing concentrations of Flag-Cdh1 (0, 0.25, 0.5, 1 µg). Band intensity was estimated using ImageJ software, normalized with GAPDH, and represented graphically. Data were presented as the mean and standard deviation of three independent experiments. One-way ANOVA followed by Tukey’s post hoc test was used, and *p*-values are indicated. (**C**) Effect of overexpression of Cdh1 on the ubiquitination and proteasomal degradation of HA-PAH was determined. HEK293 cells were transfected with HA-PAH, Flag-ubiquitin, and Flag-Cdh1 and immunoprecipitated with HA antibody and immunoblotted with indicated antibodies.

To identify the E3 ligase that directs the ubiquitin-proteasome system (UPS)-mediated degradation of PAH, we screened a panel of E3 ligases and checked the corresponding PAH level in HEK293 cells. Flag-TRCP1, Flag-TRCP2, HA-Cbl/Grb2, and Flag-Cdh1 were co-transfected along with HA-PAH in HEK293 cells and the level of the PAH expression was analyzed by Western blotting. Western blot analysis indicated that from several E3 ligases, Flag-Cdh1 significantly degraded PAH (Figure 2A, lane 5). To further validate our findings, we investigated the impact of overexpression of Flag-Cdh1 in a dose dependent-manner on exogenous PAH protein. HEK293 cells were co-transfected with a constant amount of HA-PAH along with increasing concentrations of Flag-Cdh1. We observed a dose-dependent decrease in PAH levels upon a dose-dependent increase in Cdh1 suggesting that Cdh1 destabilizes and degrades PAH protein (Figure 2B).

Furthermore, the ubiquitination status of PAH was analyzed in the presence of Cdh1 overexpression. For that, we co-transfected HEK293 cells with HA-PAH, Flag-ubiquitin, and Flag-Cdh1 and performed immunoprecipitation analysis with the antibodies indicated. A high intensity ubiquitination smear and reduced PAH expression was observed in samples transfected with Flag-Cdh1 along with HA-PAH and Flag-ubiquitin (Figure 2C, lane 4) compared to samples transfected with only HA-PAH and Flag-ubiquitin (Figure 2C, lane 3). Thus, these results indicate that APC/C^Cdh1^ is an E3 ligase for PAH and it is responsible for ubiquitination and rapid degradation of PAH by the 26S proteasome system.

### 2.3. APC/C^Cdh1^ Is a Negative Regulator of PAH Protein Stability

**Figure 3:**
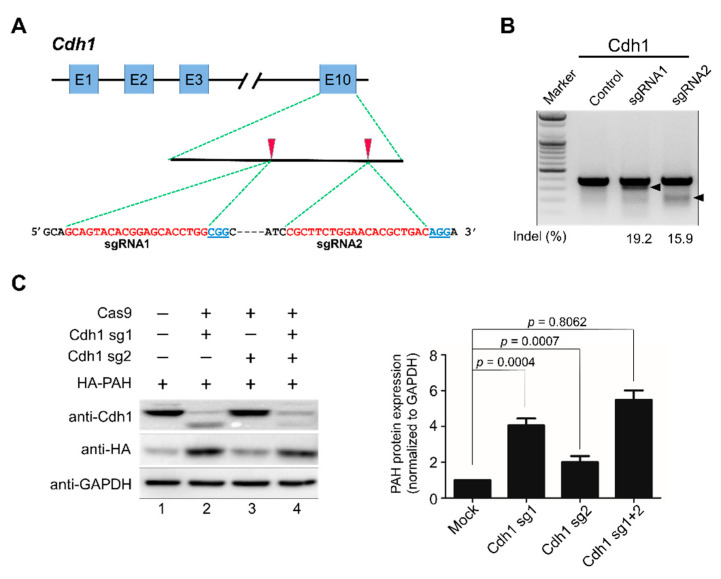
APC/C^Cdh1^ is a negative regulator of PAH stability. (**A**) Schematic of the RNA-guided engineered nuclease targeting the human *Cdh1* gene with designed sgRNA1 and sgRNA2 that target sequences in exon 10. Blue boxes represent exons. Red arrowheads indicate the positions of sgRNAs targeting the top strand. Target sequences are represented in red and PAM sequences are marked by bold blue underlined font. (**B**) The cleavage efficiency of sgRNA1 and sgRNA2 was determined by the T7E1 assay in HEK293 cells after transfection with plasmids encoding Cas9 and sgRNAs. The size marker is shown. Arrows indicate the expected positions of the cleaved DNA bands. The numbers at the bottom of the gel indicate indel percentage measured by band intensity using ImageJ software. Untransfected cells were used as a negative control. (**C**) The knockout efficiency of sgRNAs targeting *Cdh1* was determined by Western blot analysis HEK293 cells were transiently transfected with two sgRNAs targeting *Cdh1* along with ectopically expressing HA-PAH to check exogenous protein levels. Band intensity was estimated using ImageJ software, normalized with GAPDH, and represented graphically. Data were presented as the mean and standard deviation of three independent experiments. One-way ANOVA followed by Tukey’s post hoc test was used, and *p*-values are indicated.

To better understand the effect of Cdh1 on PAH turnover, a clustered regularly interspaced short palindromic repeats/CRISPR-associated-9 (CRISPR/Cas9) system-based knockout of *Cdh1* was performed. We individually designed two sets of sgRNA targeting *Cdh1*. The two sgRNAs targeting the late exonic (exon 10) region of *Cdh1* are represented in Figure 3A. To ensure high efficiency and specificity, sgRNAs were designed based on the scoring system obtained from the Genetic Perturbation Platform (GPP) sgRNA designer (www.broadinstitute.org). The efficiencies of sgRNAs were validated based on the high indel percentage obtained from the T7E1 assay. The sgRNA-1 targeting the *Cdh1* gene showed a higher indel percentage than sgRNA-2 (Figure 3B). Then, we transiently transfected two sgRNAs targeting *Cdh1* separately or combined and analyzed the expression of Cdh1 protein and its effect on PAH expression. The results showed that sgRNA-1 targeting *Cdh1* significantly decreased Cdh1 expression and proportionally increased PAH expression (Figure 3C, lane 2). However, the sgRNA-2 that showed a low T7E1 efficiency (Figure 3B) had no significant effect on reducing Cdh1 expression (Figure 3C, lane 3). Additionally, the transfection of both sgRNAs exhibited a similar effect on Cdh1 and PAH expression as showed by sgRNA-1 alone (Figure 3C, lane 4). This result is in line with the high indel percentage observed in sgRNA-1 as compared to sgRNA-2 targeting the *Cdh1* gene (Figure 3B). Thus, sgRNA-1 with a high efficiency was considered for further functional assays.

### 2.4. APC/C^Cdh1^ Interacts and Co-Localizes with PAH

**Figure 4:**
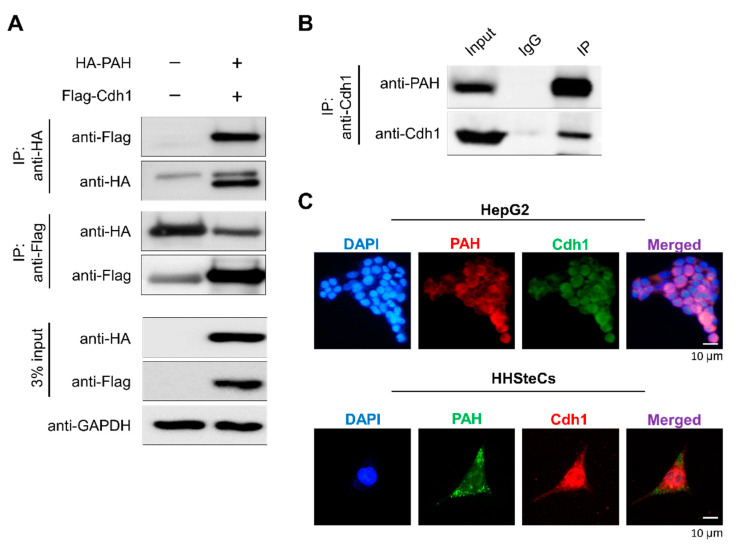
APC/C^Cdh1^ interacts and co-localizes with PAH. (**A**) HA-PAH and Flag-Cdh1 were co-transfected into HEK293 cells. Samples were immunoprecipitated using respective antibodies and immunoblotted using indicated antibodies. GAPDH was used as a loading control. (**B**) Endogenous interactions between PAH and Cdh1 proteins were conducted in HHSteCs. Samples were immunoprecipitated using specific anti-Cdh1 antibody and immunoblotted using specific PAH antibody. (**C**) Immunostaining with PAH and Cdh1 antibodies was performed to analyze endogenous localization patterns in HepG2 and HHSteCs. DAPI was used as a nuclear stain. (Scale bar: 10 µm).

To analyze the role of Cdh1 in regulating PAH in cells, we first checked the physical association between the two proteins in vivo. We co-transfected HA-PAH and Flag-Cdh1 in HEK293 cells and performed immunoprecipitation with HA or Flag antibodies, followed by Western blotting with reciprocal antibodies. The results showed that HA-PAH co-precipitated with Flag-Cdh1 and vice versa (Figure 4A). Furthermore, we assessed the association between PAH and Cdh1 at the endogenous level. The endogenous co-immunoprecipitation studies in Human hepatic stellate cells (HHSteCs) revealed that Cdh1 interacts with PAH within cells (Figure 4B). Thus, co-immunoprecipitation studies demonstrate that Cdh1 is a binding partner of PAH at both the exogenous and endogenous levels.

To shed light on the spatial control of Cdh1 activity on PAH, we investigated the subcellular localization by immunofluorescence of both the proteins at the endogenous level in HHSteCs and HepG2 cells. Consistent with previous reports, PAH was found predominantly in the cytoplasm, while Cdh1 is localized in both cytoplasm as well as the nucleus [14,15] (Figure 4C).

### 2.5. APC/C^Cdh1^ Declines PAH Protein Half-Life

**Figure 5:**
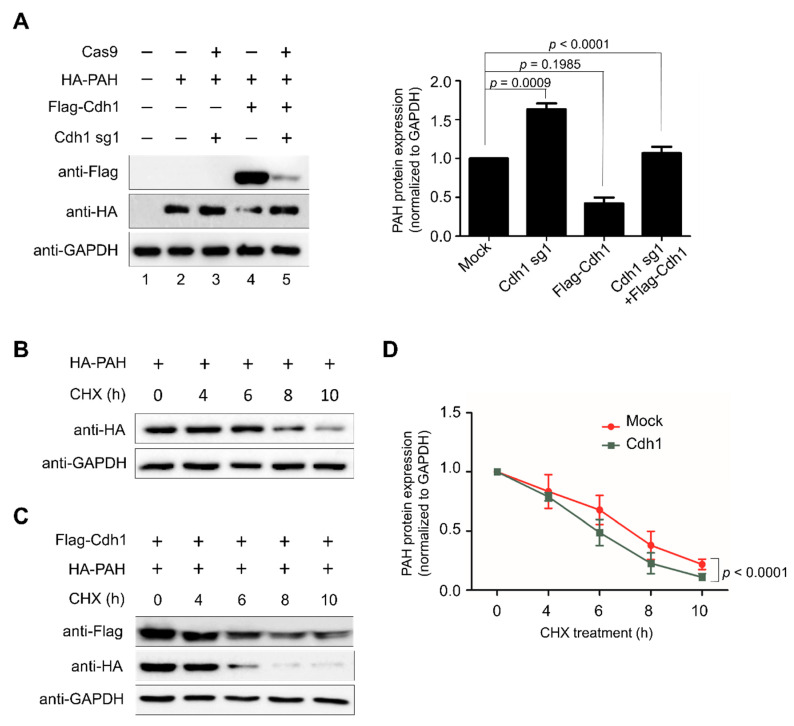
APC/C^Cdh1^ declines PAH protein half-life. (**A**) HA-PAH protein degradation mediated by Cdh1 was rescued upon depleting Cdh1. Protein expression was detected through indicated antibodies and analyzed by Western blotting. Band intensity was estimated using ImageJ software, normalized with GAPDH, and represented graphically. Data were presented as the mean and standard deviation of three independent experiments. One-way ANOVA followed by Tukey’s post hoc test was used, and *p*-values are indicated. (**B**) HEK293 cells were transfected with HA-PAH and treated with cycloheximide (CHX, 150 µg/mL), harvested at different time points, and analyzed by Western blotting with the antibodies indicated. Band intensity was estimated using the ImageJ software, normalized with GAPDH, and graphically represented graphically. (**C**) HEK293 cells were transfected with HA-PAH in combination with Flag-Cdh1 and treated with cycloheximide (CHX, 150 μg/mL), harvested at different time points, and analyzed by Western blotting with the antibodies indicated. (**D**) Band intensity of HA-PAH from both (**B**,**C**) was estimated using ImageJ software, normalized with GAPDH, and represented graphically. Data were presented as the mean and standard deviation of three independent experiments. Two-way ANOVA followed by Tukey’s post hoc test was used, and *p*-values are indicated.

To check the role of Cdh1 on PAH turnover, we performed both overexpression or knockout of Cdh1 and analyzed the PAH level. HEK293 cells were transiently transfected with Flag-Cdh1 or sgRNA-1 targeting *Cdh1* along with ectopically expressed HA-PAH. The results indicated that the cells transfected with Flag-Cdh1 along with HA-PAH had reduced levels of PAH compared to the control sample (Figure 5A, lane 4). However, Cdh1-mediated protein degradation of PAH was reversed when cells were co-transfected with sgRNA-1 targeting *Cdh1* along with Flag-Cdh1 and HA-PAH (Figure 5A, lane 5) when compared to samples transfected with only Flag-Cdh1 and HA-PAH (Figure 5A, lane 4). Taken together, these findings indicate that APC/C^Cdh1^ is a specific E3 ligase regulating the steady-state levels of PAH in cells.

Previous reports suggest that the average half-life of PAH is approximately 7–8 h [16,17]. To analyze the effect of post-translational modification mediated by APC/C^Cdh1^ on the PAH protein half-life, we performed a cycloheximide chase assay. Upon cycloheximide treatment, PAH protein showed half-life of about 8 h (Figure 5B), while overexpression of Cdh1 promoted rapid degradation of PAH and declines the half-life of PAH (Figure 5C). These data suggest that APC/C^Cdh1^ is a critical regulator of PAH turnover which declines the half-life of PAH protein from 8 h to 6 h (Figure 5D).

### 2.6. APC/C^Cdh1^ Promotes PAH Polyubiquitination

**Figure 6:**
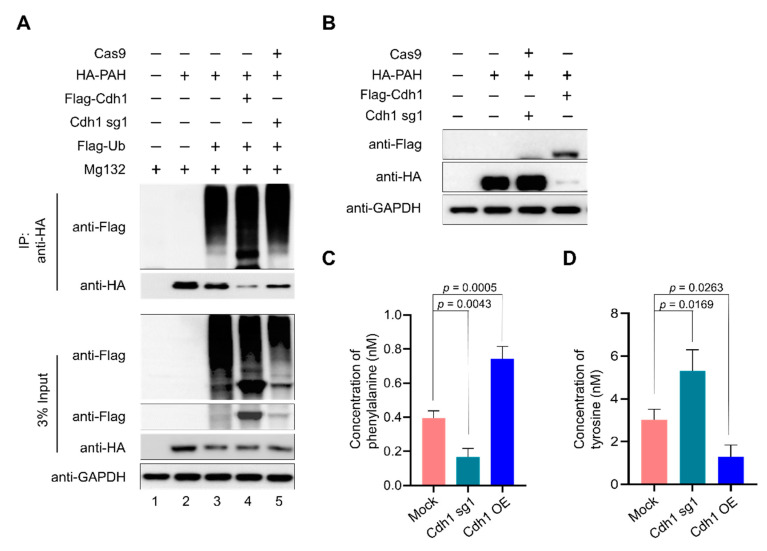
APC/C^Cdh1^ promotes phenylalanine metabolism. (**A**) HEK293 cells were transfected with HA-PAH alone or with Flag-ubiquitin, Flag-Cdh1, or sgRNA1 targeting *Cdh1*. The ubiquitination of PAH was confirmed by co-immunoprecipitation with anti-HA antibody and immunoblotted with the antibodies indicated. (**B**) The transfection efficiency of respective constructs was confirmed by Western blot analysis. (**C**) Effect of Cdh1 depletion on phenylalanine metabolism using a phenylalanine assay kit. (**D**) Effect of Cdh1 depletion on phenylalanine metabolism using a Tyrosine assay kit. Data are presented as the mean and standard deviation of three independent experiments. One-way ANOVA followed by Dunnett’s test was used and *p*-values are indicated.

To assess the effect of Cdh1 on the ubiquitination of PAH, we analyzed the polyubiquitination status of PAH in the presence of Cdh1 overexpression and sgRNA1 targeting *Cdh1* in HEK293 cells. HA-PAH, Flag-Cdh1, and sgRNA targeting *Cdh1* were transfected along with Flag-ubiquitin in HEK293 cells treated with MG132 for 8 h and immunoprecipitation was performed using anti-HA, followed by Western blot analysis with respective antibodies (Figure 6A). Overexpression of Cdh1 promoted UPS-mediated degradation of PAH, which is evident by the intense ubiquitin smear observed in Flag-Cdh1 transfected samples along with Flag-ubiquitin and HA-PAH constructs (Figure 6A, lane 4) as compared with only Flag-ubiquitin and HA-PAH transfected cells (Figure 6A, lane 3). The ubiquitin smear was reduced upon knockout of *Cdh1* using sgRNA-1 and Cas9 (Figure 6A, lane 5). Thus, the aforementioned data indicate that APC/C^Cdh1^ promotes polyubiquitination of the PAH protein leading to rapid degradation.

### 2.7. Depletion of APC/C^Cdh1^ Promotes Phenylalanine Metabolism

Toxic accumulation of phenylalanine in the blood is one of the hallmarks of patients with PKU. Here, we wished to investigate the role of APC/C^Cdh1^ in regulating PAH enzymatic activity. For this purpose, we transfected HA-PAH, Flag-Cdh1, and sgRNA targeting *Cdh1* in 293 cells and checked the expression of PAH by Western blot (Figure 6B). The same samples were subjected to PAH activity assay to quantify the amount of l-Phe, which is the substrate, and l-Tyr, which is the product, in the phenylalanine metabolism pathway in the presence and absence of Cdh1. We observed high hydroxylation of l-Phe to l-Tyr in Cdh1 depleted cells when compared with overexpressed Cdh1 samples resulting in less accumulation of l-Phe in Cdh1 depleted cells (Figure 6C). Similarly, we observed a high amount of l-Tyr in Cdh1 depleted cells when compared with overexpressed Cdh1 samples (Figure 6D). Our results signify that PAH-mediated metabolism of phenylalanine to tyrosine is significantly increased in Cdh1 depleted cells, which is evident by the amount of substrate l-Phe metabolized and l-Tyr produced in the cells when compared with the mock control (Figure 6C,D).

### 2.8. Association of APC/C^Cdh1^ and PAH in HCC

**Figure 7:**
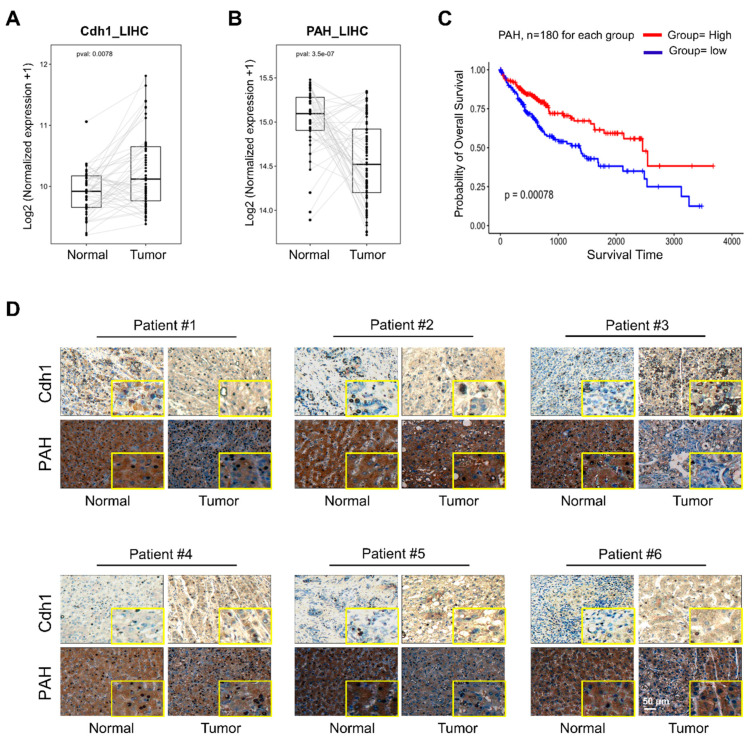
Association of APC/C^Cdh1^ and PAH in HCC. (**A**) Expression box plot showing significant differential expression of *Cdh1* in normal vs. tumor tissues using normal-tumor matched liver cancer patient RNAseq data from TCGA. *p* value < 0.05 was considered to be statistically significant. (**B**) Expression box plot showing significant differential expression of *PAH* in normal vs. tumor tissues using normal-tumor matched liver cancer patient RNAseq data from TCGA. *p* value < 0.05 was considered to be statistically significant. (**C**) Kaplan–Meier survival curves. Liver cancer patients were divided into two groups based on PAH expression pattern, and the overall survival data from the high and low groups were used for the Kaplan–Meier analysis. *p* value < 0.05 was considered to be statistically significant. (**D**) Representative immunohistochemical staining of endogenous PAH and Cdh1 in human liver (Tumor) and non-neoplastic tissue (Normal) (inset shows location of threefold magnified regions) Scale bar = 50 µm.

The UPS has been increasingly recognized as a critical factor regulating normal cellular function and a potential target during cancer progression and survival [18]. Previous reports suggest that E3 ligases such as Parkin and SIAH1 are involved in HCC [19,20,21,22]. Interestingly, APC/C^Cdh1^ is known to behave as both tumor suppressor and oncoprotein in various cancer types [23,24,25,26,27]. After demonstrating the interaction between Cdh1 and PAH, we wished to investigate the expression correlation between Cdh1 and PAH in HCC. The Human Protein Atlas database suggested that the protein expression of PAH was detected only in few tissues such as liver, kidney, and gallbladder (Figure S1A). Considering *PAH* as a liver gene, we analyzed the expression levels of Cdh1 and PAH individually using The Cancer Genome Atlas (TCGA) data in liver hepatocellular carcinoma (LIHC) for which normal-tumor matched RNAseq expression data were available. Cdh1 was significantly upregulated in tumors tissues when compared with normal tissues (Figure 7A). In contrast, PAH was significantly downregulated in tumor tissues than in normal tissues showing a possible regulatory link between the expression levels of Cdh1 and PAH (Figure 7B).

In order to assess prognostic significance of PAH in LIHC, we correlated PAH-expression status with the patient’s survival data. To support our data, Kaplan–Meier analysis of TCGA survival data showed that the patient group with high expression of PAH in LIHC showed prolonged overall survival (Figure 7C). Furthermore, immunohistochemistry (IHC) staining of 12 liver cancer tissues obtained from the ISU ABXIS cohort showed that Cdh1 was predominantly expressed in the cytoplasm of hepatocarcinoma cells and normal hepatocytes; and the expression level of Cdh1 was significantly upregulated in HCC tissues than in the non-neoplastic tissues (Figure 7D, upper panel, Figure S1B). On the contrary, a moderate expression of PAH was observed in patient tumor tissue samples compared with high expression in matched non-neoplastic tissues (Figure 7D, lower panel, Figure S1B). Thus, our PAH expression and overall survival studies indicate that the higher expression of PAH was associated with a good clinical outcome suggesting a favorable prognosis effect of PAH and plays a protective role in prognosis of HCC. As developing a specific prognostic marker for HCC is still a challenge, our study demonstrates that PAH could be a novel therapeutic target in the early prognosis of HCC.

## 3. Discussion

PAH is a highly regulated liver enzyme that catalyzes the rate-limiting step in phenylalanine and tyrosine metabolism [5]. PAH is responsible for the oxidation of excess l-Phe into carbon dioxide and water and degrades about 75% of the l-Phe in the diet [28]. In 1934, Asbjørn Følling was the first to detect elevated levels of phenylketonuric acid in the urine of two siblings with mental impairment and named it “phenylpyruvic oligophrenia” or PKU [7]. Several mutations in the *PAH* gene were detected in PAH deficient PKU patients from different ethnic populations [29,30,31]. Additionally, several studies have demonstrated covalent and non-covalent regulation of its enzyme kinetics [10,32]. Moreover, mutations in *PAH* lead to protein instability, misfolding, rapid degradation, and deficiency in enzymatic activity [33,34,35].

To combat the toxic accumulation of mutated proteins that are prone to misfolding, cells are facilitated with a protein quality control system such as molecular chaperones and the UPS. Several molecular chaperones in the heat shock protein (HSP) family recognize these misfolded proteins and coordinates with the UPS for protein refolding and/or degrading of misfolded proteins [36]. Importantly, E3-chaperone complexes work in parallel to target misfolded proteins for degradation. The E3 ligase Hsc70 interacting protein regulates the function of Hsp70/Hsp90 to coordinate cellular protein folding and degradation [37]. Thus, we initiated this study to find specific E3 ligases that regulate the ubiquitination and degradation of PAH to understand the molecular mechanism of PAH protein dynamics at the physiological and pathological levels.

In this study, we demonstrate the APC/C^Cdh1^ E3 ligase-dependent proteolysis of PAH. APC/C is a multisubunit E3 ligase that polyubiquitinates target substrates for degradation. APC/C is activated in a cell cycle-dependent manner by WD-40 domain proteins, namely CDC20 and Cdh1 [38]. In the early stages of mitosis, APC/C^Cdc20^ regulates the initiation of anaphase while APC/C^Cdh1^ covers an array of substrates involved in and beyond the cell cycle [39]. In Alzheimer’s disease and stroke pathogenesis, APC/C^Cdh1^, but not APC/C^Cdc20^, is expressed in post-mitotic mammalian neurons, and the inactivation of Cdh1 by phosphorylation results in the accumulation of cyclin B1 [40]. Thus, we expanded our study to investigate the regulatory role of APC/C^Cdh1^ on PAH expression and its effect on phenylalanine metabolism. Here, we demonstrate that APC/C^Cdh1^ recognizes and subjects PAH to 26S proteasomal degradation (Figure 1A–C). There are several lysine (K)-linked ubiquitin chains such as K6, K11, K27, K29, K33, K48, and K63 attached to the substrate and mark them for proteasomal degradation [41]. Among them, K48- and K63-linked chains are well studied and target the substrate for proteasomal degradation [42,43,44]. Additionally, chains generated via K63-linkage exhibits various non-proteolytic roles in cell signaling and DNA repair pathways [45]. In our study, we demonstrate that PAH undergoes both K48- and K63-linked polyubiquitination (Figure 1D). The overexpression of APC/C^Cdh1^ negatively regulates PAH protein levels (Figure 2A,B), which is also evident by the higher ubiquitination of PAH in the presence of Cdh1 overexpression (Figure 2C, lane 4). We also showed that the levels of PAH were significantly increased upon the *CRISPR/Cas9* mediated knockout of *Cdh1* (Figure 3). Furthermore, we demonstrate that Cdh1 physically interacts with the PAH protein in vivo and co-localizes in the nucleus/cytoplasm (Figure 4). As a functional consequence of the interaction between Cdh1 and PAH, Cdh1 declines the half-life of PAH (Figure 5) and subsequently lowers the availability of the protein for phenylalanine metabolism. Thus, we have demonstrated that the depletion of Cdh1 significantly increases PAH levels to facilitate the hydroxylation of l-Phe to l-Tyr (Figure 6C,D).

The expression and localization of E3s is often altered in human malignancies due to accumulation of mutations, specific post translational modification converting them from a tumor suppressor to a tumor promoter and vice versa [46]. There are evidences suggesting that E3s can switch their function as tumor suppressive or oncogenic depending on its target substrate, subcellular localization, and biological context. For example, peckle-type pox virus and zinc finger (POZ) protein (SPOP), a representative substrate-recognition subunit of the cullin-RING E3 ligase, is known to play a dual role as an oncogene in the cytoplasm but as tumor suppressor in the nucleus [47,48]. Also, SMURF2 and Mdm2 that acts as a potent tumor suppressor in normal cells, can behave as an oncogene in established tumors [49,50]. Therefore, E3 ligases mark as attractive disease biomarkers; however, it is important to understand the complexity of its function and expression in a tissue- and tumor-dependent manner. Several bodies of evidence have determined that E3 ligases are associated with HCC [18,51]. The E3 ligase SIAH1 is significantly downregulated in HCC whereas TRIM31 was found to upregulated promoting the malignant behavior of HCC cells [19,52]. There are evidence supporting the tumor suppressive role of E3 ligase Parkin in HCC. There is enhanced hepatocyte proliferation with a high frequency of macroscopic liver tumor development in Parkin-deficient transgenic mice [20,21,22]. Another report suggests the involvement of the 26S proteasome subunit Gankyrin in early HCC pathogenesis [53].

Since HCC is a leading cause of cancer mortality and is estimated to be the fourth most common cancer worldwide [54,55,56], exploring the possible roles of UPS in the disease is essential. Although several genetic alterations are associated with the pathogenesis of HCC, information about the contribution of E3 ligases in this process is limited. Therefore, we analyzed the association of APC/C^Cdh1^ and PAH with high-grade tumors and prognosis in HCC. Here, by using in silico analysis, we demonstrated that *Cdh1* was upregulated while *PAH* was significantly downregulated in HCC indicating a negative correlation between Cdh1 and PAH (Figure 7A,B). Finally, our hypothesis was well supported by clinical studies that have demonstrated low PAH levels in the tumor tissue samples of most patients compared with normal tissue samples (Figure 7D). Therefore, our study provides a new insight about the expression pattern of Cdh1 and PAH in liver cancer tissues indicating its possible role in the progression of HCC.

Although our study highly suggests the possibility of PAH as a prognostic marker and demonstrates its negative correlation with Cdh1 in HCC, further validation is needed to elucidate the involved molecular mechanism. Moreover, the correlation between PAH and Cdh1 in cancer progression and its potential application in cancer therapy should be performed in the future to elucidate its molecular mechanism during the progression of HCC.

## 4. Conclusions

In conclusion, our study represents the first attempt to identify an E3 ligase that regulates the stability and turnover of PAH. This study identifies APC/C^Cdh1^ as an E3 ligase that promotes polyubiquitination of PAH resulting in the reduction of the PAH protein pool for phenylalanine metabolism. Thus, we suggest that the knockout of the *Cdh1* gene enhances the hydroxylation of phenylalanine to tyrosine. Additionally, we have demonstrated that the expression of *Cdh1* was high and *PAH* was low in HCC tissues. Moreover, the high expression of PAH was associated with good clinical outcome showing prolonged overall survival. Thus, we propose that Cdh1 could be a potential therapeutic target to regulate PAH-mediated physiological disorders.

## 5. Materials and Methods

### 5.1. Plasmids

Mammalian expression vector encoding HA- and GFP-tagged PAH were kindly provided by Prof. Shen Nan (University Children’s Hospital, Heidelberg, Germany), and Myc-PAH was kindly provided by Prof. Lourdes Ruiz Desviat (Autonomous University of Madrid, Madrid, Spain). Flag-tagged Grb2, Flag-tagged ubiquitin, and HA-tagged Cbl were kindly provided by Prof. Yun Soo Bae (Ewha University, Seoul, Korea); Flag-tagged Cdh1, Flag-tagged TRCP1, and Flag-tagged TRCP2 were kindly provided by Prof. Zhao Qi Wang (Leibniz Institute on Aging-Fritz Lipmann Institute (FLI), Jena, Germany). HA-tagged ubiquitin (Cat no. #18712), HA-tagged-ubiquitin-K48 (Cat no. #17605), and HA-tagged-ubiquitin-K63 (Cat no. #17606) were purchased from Addgene, Watertown, Massachusetts, USA.

### 5.2. Antibodies and Reagents

Mouse monoclonal antibodies against PAH (sc-271258, 1:50), Flag (Anti-DDDDK-tag, M185-3L, 1:1000) (MBL Life Science, Woburn, MA, USA), ubiquitin (sc-8017, 1:1000), HA (sc-7392, 1:1000), GAPDH (sc-32233, 1:1000), and normal mouse IgG (sc-2025, 1:1000) were purchased from Santa Cruz Biotech, Dallas, TX, USA. PAH (MAB5278, 1:1000, Merck Millipore, Kenilworth, N.J., USA), rabbit polyclonal antibodies against FZR1/Cdh1 (Cat no. #34-2000, 1:1000 Invitrogen, Carlsbad, CA, USA), and 488/594-conjugated secondary antibodies (Cat no. #A21207, Cat no. #A21203, 1:200) (Life Technologies, Carlsbad, CA, USA) were also used. In addition, we used Protein A/G Plus Agarose beads (sc-2003, Santa Cruz Biotech, Dallas, TX, USA), proteasomal inhibitor MG132 (Cat no. #S2619, Selleckchem, Houston, TX, USA), protein translation inhibitor cycloheximide (CHX; Cat no. #239765, Merck, Kenilworth, NJ, USA), and protease inhibitor cocktail (Cat no. #B14012, Bimake.com, Korea).

### 5.3. Cas9 and sgRNA Constructs

For the screening of single-guide RNAs (sgRNAs), a plasmid encoding Cas9-2a-mRFP-2a-PAC (puromycin N-acetyl-transferase, puromycin resistance gene) and plasmid encoding sgRNAs were purchased from Toolgen (Seoul, Korea). The sgRNA target sequences were based on bioinformatics tools (www.broadinstitute.org) and cloned into the vectors as described previously [57]. Briefly, oligonucleotides containing each target sequence were synthesized (Bioneer, Seoul, Korea) and T4 polynucleotide kinase was used to add terminal phosphates to the annealed oligonucleotides (BioRad, Hercules, CA, USA). The vector was digested with *Bsa*I restriction enzyme and ligated with annealed oligonucleotides. Oligonucleotide sequences are listed in Supplementary Table S1.

### 5.4. Cell Culture and Transfections

Human embryonic kidney (HEK293) cells were cultured in DMEM (GIBCO BRL, Rockville, MD, USA) supplemented with 10% Fetal bovine serum (GIBCO BRL, Rockville, MD, USA) and 1% penicillin and streptomycin (GIBCO BRL, Rockville, MD, USA) at 37 °C in a humidified atmosphere with 5% CO_2_. HHSteCs were kindly provided by Yun Soo Bae (Ewha University, Seoul, Korea) and cultured in DMEM supplemented with 10% fetal bovine serum and 1% penicillin and streptomycin at 37 °C in a humidified atmosphere with 5% CO_2_. The cells were passaged every 2–4 days depending on cell confluence. HEK293 and HHSteCs were used for all exogenous and endogenous experiments, respectively.

For transient transfection, HEK293 cells and HHSteCs were transfected with plasmids using polyethyleneimine (PEI; Polysciences, Warrington, PA, USA) according to the manufacturer’s protocol. For the knockout of *Cdh1*, HHSteCs were transfected with Cas9 and sgRNA targeting *Cdh1*. After 24 h, the transfected cells were selected by incubating with puromycin (1 µg/mL) for 48 h and then passaged before use. The puromycin selected cells with a high reduction in Cdh1 protein expression were used for functional assays. For ubiquitination and endogenous interaction assays, the cells were treated with 20 µM MG132 for 8 h before harvesting.

### 5.5. T7 Endonuclease I (T7E1) Assay

T7E1 assays were performed as described previously [58,59]. After isolation of genomic DNA using DNeasy Blood & Tissue kits (Qiagen, Hilden, Germany) according to the manufacturer’s instructions, the region of DNA containing the nuclease target site was PCR-amplified using Hemi-nested primers. The oligonucleotide sequence information to obtain PCR amplicons for the T7E1 assay and the expected cleavage sizes after the T7E1 assay are mentioned in Supplementary Tables S2 and S3. Amplicons were denatured by heating and then annealed to form heteroduplex DNA, which was treated with 5 units of T7 endonuclease 1 (New England Biolabs, Ipswich, Massachusetts, United States) for 15 to 20 min at 37 °C and analyzed using 1.5% agarose gel electrophoresis. Mutation frequencies were calculated based on band intensity using ImageJ software and the equation: mutation frequency (%) = 100 × (1 − [1 − fraction cleaved] × 1/2), where fraction cleaved was the total relative density of the cleavage bands divided by the sum of the relative density of cleavage and uncut bands.

### 5.6. Immunoprecipitation and Immunoblotting

For binding and ubiquitination assay, HEK293 cells were transfected with the respective constructs mentioned in Figure 1, Figure 2, Figure 4 and Figure 6. At 48 h post-transfection, an immunoprecipitation assay was performed. Cells were lysed in buffer B containing 50 mM Tris-HCl (pH 7.6), 150 mM NaCl, 1 mM EDTA, 1% Triton X-100, and a protease inhibitor cocktail. About 3 mg of cell lysates were incubated with the antibodies indicated at 4 °C overnight. The next day, the lysate was immunoprecipitated with protein agarose beads at 4 °C for 3–4 h. Immuno-complexes were washed with lysis buffer and eluted and boiled in 2× SDS sample buffer. They were then detected by Western blot analysis and 3% of the samples were used to identify the immunoprecipitation efficiency as the whole-cell lysate. For endogenous immunoprecipitation assay, about 6 mg of lysates from HHSteCs were incubated with indicated antibodies and detected by Western blot analysis where 3% of the samples were used to identify immunoprecipitation efficiency as whole cell lysate. Mouse IgG (ab-99697) and rabbit IgG (CST-58802S) light chain-specific secondary antibodies were used to prevent interference from heavy and light immunoglobulin chains in the binding assay.

### 5.7. Immunofluorescence Microscopy

Localization studies were performed in HepG2 and HHSteCs. Cells were seeded in 4-well culture dishes and incubated at 37 °C in a humidified atmosphere with 5% CO_2_ for 36 h. After washing in PBS, cells were fixed for 15 min in 4% paraformaldehyde and permeabilized with 0.1% Triton X-100 (in PBS) for 10 min. The slides were incubated with respective primary antibodies at 4 °C overnight. After washing with PBS, the slides were incubated with 1 μg/mL Alexa Fluor 488/594-conjugated secondary antibodies and counterstained using VECTASHIELD anti-fade mounting medium with DAPI for staining. The immunofluorescence stained images were captured using a Leica fluorescence microscope (Leica, DM 5000 B; Leica CTR 5000; Wetzlar, Germany).

### 5.8. PAH Activity Assay

HEK293 cells were co-transfected with HA-PAH and Flag-Cdh1 or sgRNA targeting the Cdh1 gene. After 48 h, transfected cells were harvested using trypsin and washed with 1× PBS, pH 7.2, then processed immediately for PAH activity assay.

Cell lysates were prepared in lysis buffer (0.25 mol/L sucrose, 1× PBS, pH 7.2) containing protease inhibitor by three freeze-thaw cycles, followed by centrifugation at 15,800 g and 4 °C for 20 min [60]. The supernatant was collected for the determination of PAH enzyme activity, which was performed according to a method described previously [4]. In brief, 100 µg of cell homogenate was mixed with 0.1 mol/L Na-HEPES buffer (pH 7.0, T&I, BHE-9000, Gangwon, Korea), 2 µg catalase (C1345, Sigma, St. Louis, MI, USA), and 1 mmol/L of l-Phe (P17008, Sigma, St. Louis, MI, USA), and incubated at 25 °C for 5 min with the addition of 100 µmol/L Fe (NH_4_)_2_(SO_4_)_2_ (215406, Sigma, St. Louis, MI, USA) for the last 1 min. The reaction was started by adding 200 µmol/L of BH_4_ (T4425, Sigma, St. Louis, MI, USA) and 5 mM DTT (10197777001, Sigma, St. Louis, MI, USA) and incubated at 25 °C for 15 min. The reaction was stopped by adding 50 µL of 2% (*w*/*v*) acetic acid in ethanol. The amount of l-Phe and l-Tyr were measured by using an Abcam Phenylalanine Assay Kit (Cat no. #ab241000, Abcam, Cambridge, UK) and a Tyrosine Assay Kit (Cat no. #ab185435), respectively, following the manufacturer’s protocol.

### 5.9. Expression and Survival Analysis Based on TCGA Data

TCGA expression data sets were downloaded from the UCSC Xena website (https://xenabrowser.net/) as processed data (level 3). Gene expression comparisons between tumor and normal samples were carried out for patients with both tumor and matched normal samples. A paired t test was used to evaluate the *p* values. A Kaplan–Meier survival analysis was performed for overall survival (OS) using the R ‘survival’ package version 2.4. OS was measured as the time from diagnosis to death from any cause. The log rank test was used to evaluate the statistical significance between expression of the gene of interest in the top and bottom tertile groups of patients. A *p* value < 0.05 was considered to be statistically significant.

### 5.10. Immunohistochemistry

Clinical samples for liver cancer tissues and normal tissues were procured from AccMax Array Inc. (Cat no. #A304, ISU Abxis Co., Seoul, Korea). The formalin-fixed paraffin-embedded tissue specimens were deparaffinized and incubated with anti-PAH (1:50) or anti-Cdh1 (1:200) according to a protocol described previously [61]. The slides were counterstained with hematoxylin, dehydrated, and mounted. Digital images were captured using a Leica DM5000 B.

### 5.11. Statistics

Statistical analysis was conducted using GraphPad Prism 9 (GraphPad Software, Inc. San Diego, CA, USA) and presented as mean ± standard deviation of three independent experiments. One-way ANOVA was used to analyze the data and multiple comparisons among the groups were performed by Tukey’s post hoc test or Dunnett’s post hoc test. For comparison between two groups, two-way ANOVA was used to analyze the data. *p* < 0.05 was considered as statistically significant.

## Supplementary Materials

The following are available online at https://www.mdpi.com/1422-0067/21/23/9076/s1.

## Abbreviations

AAAHs	aromatic amino acid hydroxylases;
PAH	phenylalanine hydroxylase;
TH	tyrosine hydroxylase;
TPH1	tryptophan hydroxylase 1;
TPH2	tryptophan hydroxylase 2;
l-Phe	l-phenylalanine;
l-Tyr	l-tyrosine;
PKU	phenylketonuria;
UPS	ubiquitin-proteasome system;
ERAD	endoplasmic reticulum-associated degradation;
APC/C	anaphase-promoting complex/cyclosome;
BH4	tetrahydrobiopterin;
Cdh1	cell division cycle 20 related protein 1;
CDC20	cell division cycle 20;
sgRNA	single-guide RNA;
T7E1	T7 endonuclease I;
HEK293	human embryonic kidney 293;
CHX	cycloheximide;
PEI	polyethyleneimine
