# Supplementary material for: E3 Ubiquitin Ligase APC/CCdh1 Regulation of Phenylalanine Hydroxylase Stability and Function
Source: Int J Mol Sci. 2020 Nov 28;21(23):9076. doi: 10.3390/ijms21239076 (PMC7729981; doi:10.3390/ijms21239076)

Supplementary Figures

E3 ubiquitin ligase APC/C^{Cdh1} regulation of phenylalanine hydroxylase stability and function

Apoorvi Tyagi[1†], Neha Sarodaya[1†], Kamini Kaushal[1], Arun Pandian Chandrasekaran[1], Ainsley Mike Antao[1], Bharathi Suresh[1], Byung-Ho Rhie[1], Kye-Seong Kim[1,2]* and Suresh Ramakrishna[1,2]*

[1]Graduate School of Biomedical Science and Engineering, Hanyang University, Seoul, South Korea

[2]College of Medicine, Hanyang University, Seoul, South Korea

† These authors have contributed equally: Apoorvi Tyagi and Neha Sarodaya

***Corresponding authors**

SR (E-mail: suri28@hanyang.ac.kr, suresh.ramakris@gmail.com);

KS (E-mail: ks66kim@hanyang.ac.kr)

Table of Contents

Figure S1: A. Comparison of PAH expression in different tissue types obtained from The Human Protein Atlas. B. Representative immunohistochemical staining of endogenous PAH and Cdh1 in human liver (Tumor) and non-neoplastic tissue (Normal).

Table S1: Oligonucleotides used for sgRNA plasmid construction.

Table S2: Oligonucleotide sequences used to get PCR amplicon for T7E1 assay.

Table S3: PCR amplicon and cleavage sizes after T7E1 assay.

Figure S2. The original uncropped images for immunoblots in the main figures.

Figure S1

A.

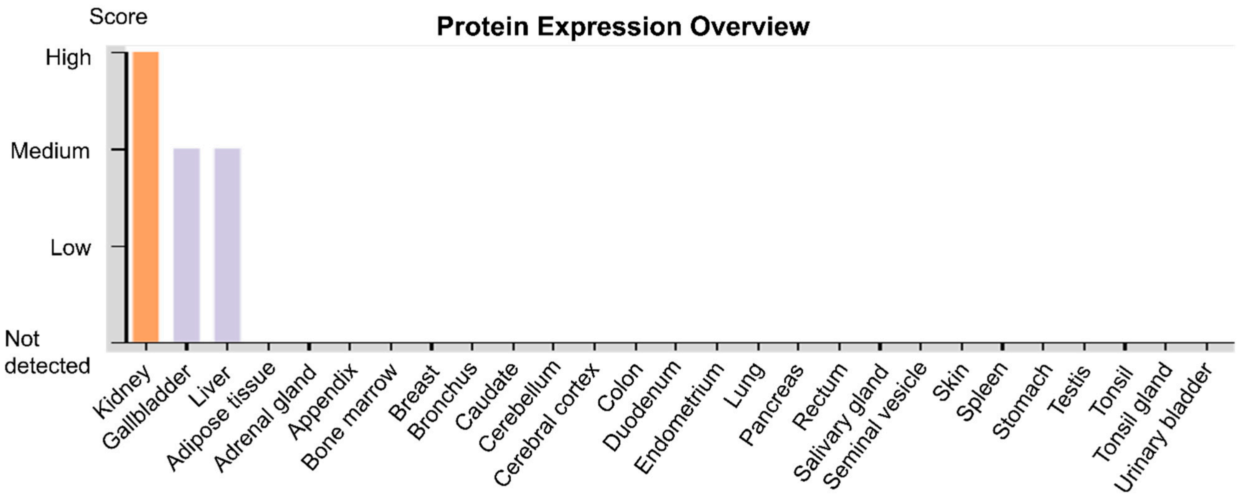
Score
Protein Expression Overview
High
Medium
Low
Not detected
Kidney
Gallbladder
Liver
Adipose tissue
Adrenal gland
Appendix
Bone marrow
Breast
Bronchus
Caudate
Cerebellum
Cerebral cortex
Colon
Duodenum
Endometrium
Lung
Pancreas
Rectum
Salivary gland
Seminal vesicle
Skin
Spleen
Stomach
Testis
Tonsil
Tonsil gland
Urinary bladder

B.

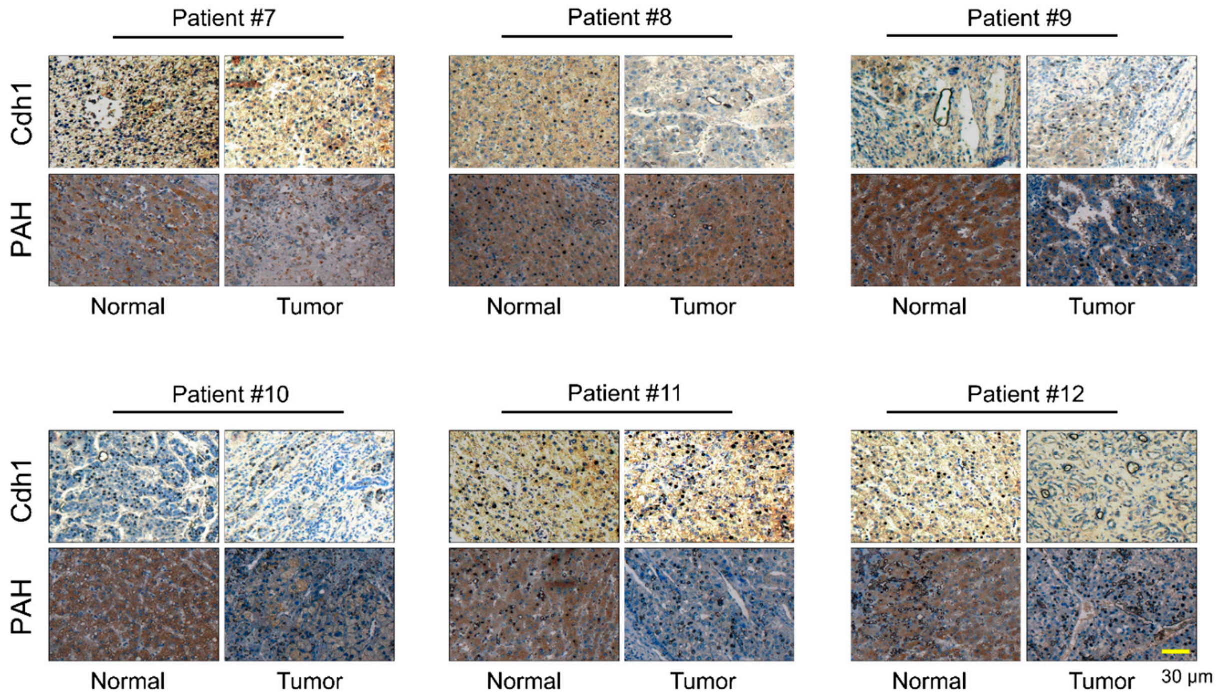
Patient #7
Patient #8
Patient #9
Cdh1
PAH
Normal
Tumor
Patient #10
Patient #11
Patient #12
30 μm

Figure S1: A. Comparison of PAH expression in different tissue types obtained from The Human Protein Atlas. Moderate to strong PAH protein expression was observed in Kidney, Liver and Gallbladder tissues. B. Representative immunohistochemical staining of endogenous PAH and Cdh1 in human liver (Tumor) and non-neoplastic tissue (Normal). Scale bar = 30 μm

Table S1: Oligonucleotides used for sgRNA plasmid construction.

Gene	sgRNA	Direction	Sequence (5' to 3')
Cdh1	sgRNA1	FP	GCAGTACACGGAGCACCTGG
		RP	CCAGGTGCTCCGTGTACTGC
	sgRNA2	FP	CGCTTCTGGAACACGCTGAC
		RP	GTCAGCGTGTTCCAGAAGCG

Table S2: Oligonucleotide sequences used to get PCR amplicon for T7E1 assay.

Gene	sgRNA	Direction	Sequence (5' to 3')
Cdh1	sgRNA1 and sgRNA2	FP	AACGACAACAAGGTACCCCC
		FP1	GCTGCTGGTCTGGAATCACT
		RP	CTATGCCTGCTGCCTCACAT
		RP1	CCATCTCCCTTCAGAGCGAC

Table S3: PCR amplicon and cleavage sizes after T7E1 assay.

Gene	sgRNA	PCR size	Cleavage size	Orientation
Cdh1	sgRNA1	355	299+56	Sense
	sgRNA2	355	151+196	Sense

Figure S2. The original uncropped images for immunoblots in the main figures.

Figure 1A

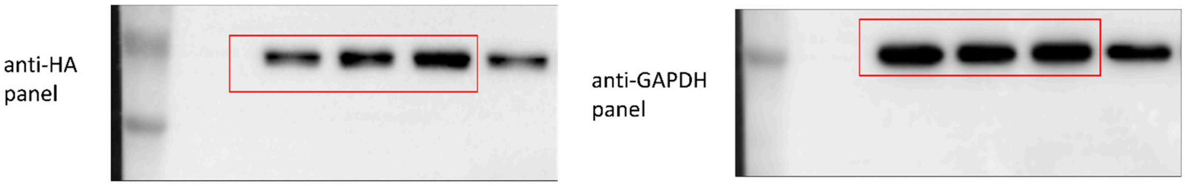

Figure 1B

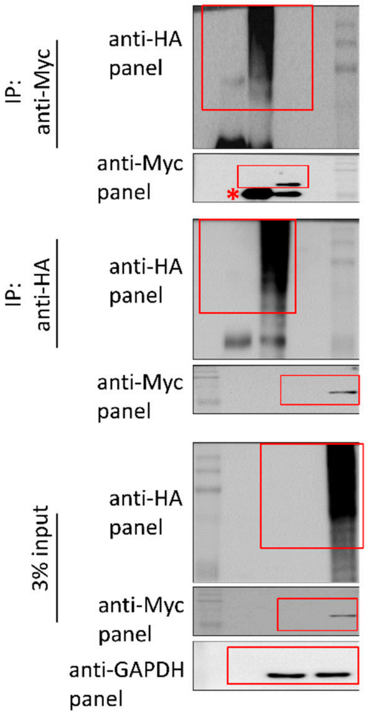

Figure 1C

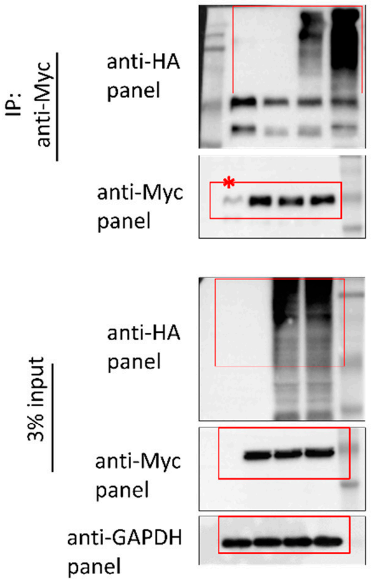

Figure 1D

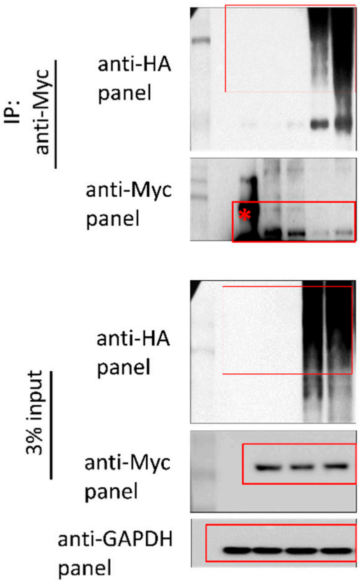

* HEAVY CHAIN

Figure 2A

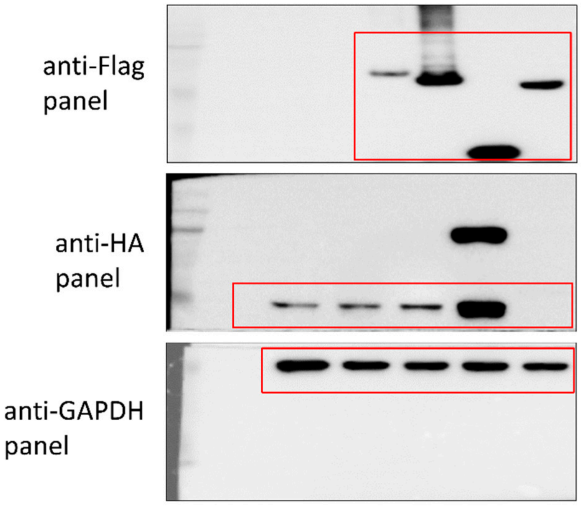

Figure 2C

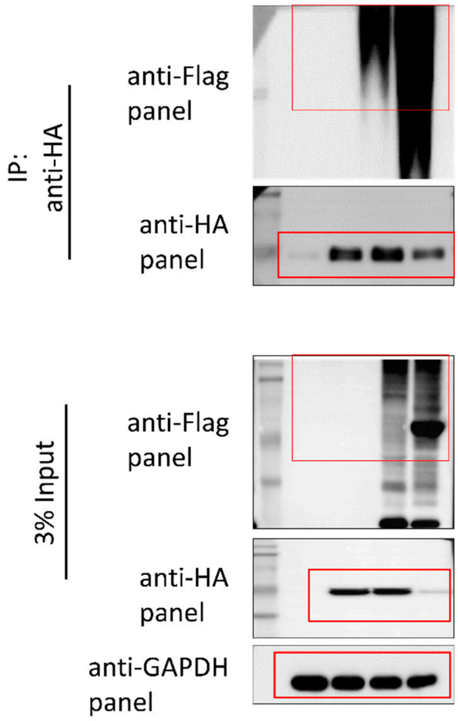

Figure 2B

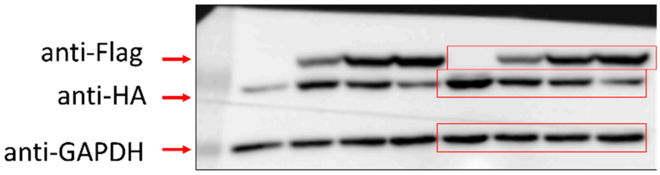

Figure 3C

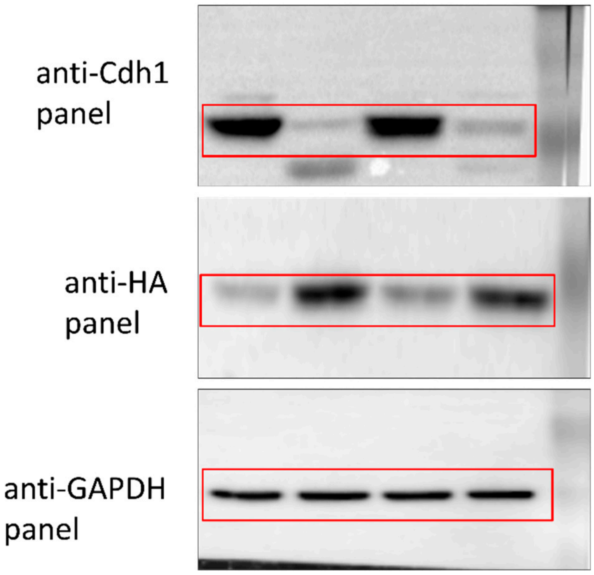

Figure 4A

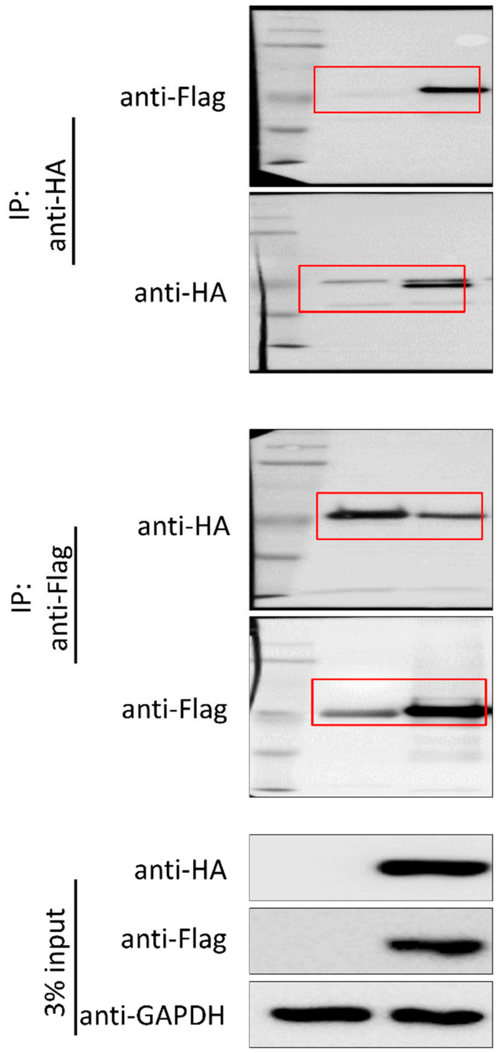

Figure 4B

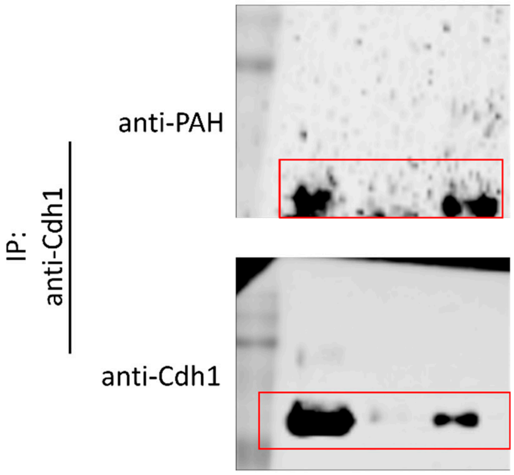

Figure 5A

anti-Flag panel

anti-HA panel

anti-GAPDH panel

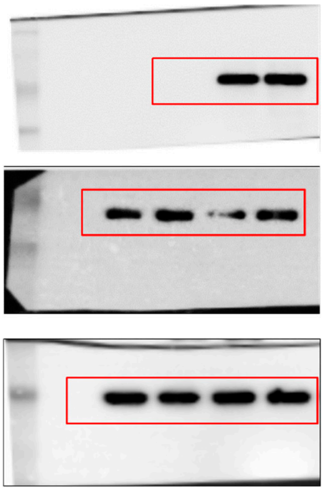

Figure 5B

anti-HA panel

anti-GAPDH panel

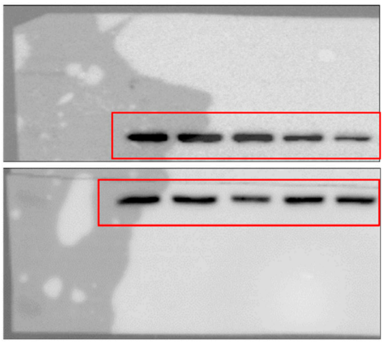

Figure 5C

anti-Flag panel

anti-HA panel

anti-GAPDH panel

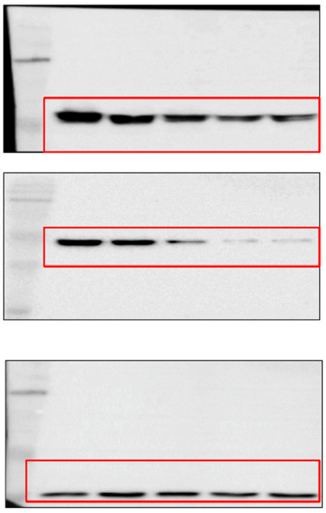

Figure 6A

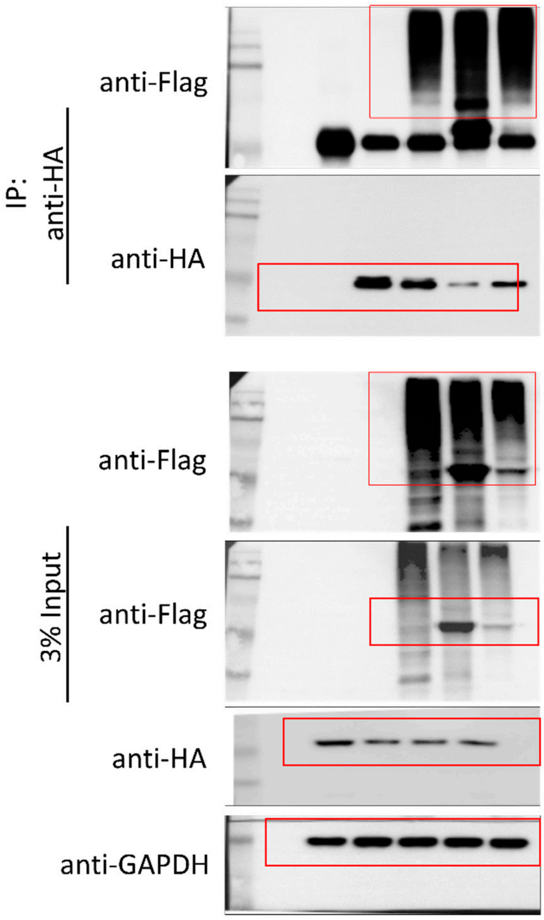

Figure 6B

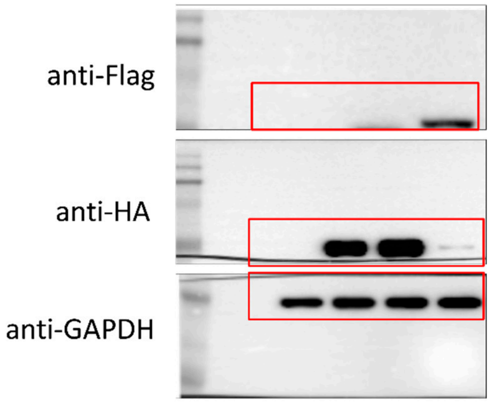

Supplement: Supplementary file 1 [file ijms-21-09076-s001.pdf]
